# Long Noncoding RNA (lncRNA) CTTN-IT1 Elevates Skeletal Muscle Satellite Cell Proliferation and Differentiation by Acting as ceRNA for *YAP1* Through Absorbing miR-29a in Hu Sheep

**DOI:** 10.3389/fgene.2020.00843

**Published:** 2020-08-07

**Authors:** Tianyi Wu, Shanhe Wang, Lihong Wang, Weibo Zhang, Weihao Chen, Xiaoyang Lv, Yue Li, Zahid Hussain, Wei Sun

**Affiliations:** ^1^College of Animal Science and Technology, Yangzhou University, Yangzhou, China; ^2^Joint International Research Laboratory of Agriculture and Agri-Product Safety of Ministry of Education of China, Yangzhou University, Yangzhou, China; ^3^Jiangsu Co-innovation Center for Important Animal Infectious Diseases and Zoonoses, Yangzhou University, Yangzhou, China

**Keywords:** *YAP1*, miR-29a, lncRNA, skeletal muscle, satellite cell, Hu sheep

## Abstract

Characterizing the factors that regulate the growth and development of muscle is central to animal production. Skeletal muscle satellite cells (SMSCs) provide an important material for simulating the proliferation and differentiation of muscle cells. *YAP1*, which can promote muscle growth, is closely related to the proliferation of SMSCs in Hu sheep (*Ovis aries*). In addition, some miRNAs, such as miR-541-3p, miR-142-5p, and miR-29a, can play critical roles in muscle growth by specifically binding with their target mRNAs. Meanwhile, lncRNA can competitively bind these miRNAs and reduce the regulatory effect of miRNAs on their target genes and thus play critical roles themselves in muscle growth. However, the regulatory molecular mechanism of miRNA and lncRNA on SMSC proliferation through *YAP1* remains unclear. Here, we characterized the regulatory network among *YAP1* and its targeted miRNAs and lncRNAs in Hu sheep SMSCs. The potential ncRNAs that regulate *YAP1* (miR-29a and CTTN-IT1) were predicted through multilevel bioinformatics analysis. Dual-luciferase assays, RT-qPCR, and western blots revealed that miR-29a can significantly reduce the mRNA and protein expression level by binding to a specific 3′-UTR of *YAP1* (*P* < 0.05), while CTTN-IT1 can restore the expression of *YAP1* through competitive binding to miR-29a. Furthermore, the mRNA and protein expression levels of MyoG, MyoD, and MyHC showed that miR-29a can inhibit the expression of genes related to the differentiation of SMSCs, and CTTN-IT1 can increase the expression of these same genes. Thus, miR-29a may inhibit the differentiation of SMSCs and CTTN-IT1 can restore this inhibition. The EdU staining assay indicated that excessive miR-29a can significantly reduce the proliferation ability of SMSCs (*P* < 0.05), while overexpression of CTTN-IT1 can significantly increase the proliferation of SMSCs (*P* < 0.01). CTTN-IT1 is a novel lncRNA that is a competing endogenous RNA (ceRNA) of miR-29a and can promote SMSC proliferation and differentiation by restoring the expression of *YAP1* when it is inhibited by miR-29a in Hu sheep. Overall, our findings construct a CTTN-IT1-miR-29a-*YAP1* regulatory network that will help contribute new insight into improving the muscle development of Hu sheep.

## Introduction

Hu sheep (*Ovis aries*), a well-known Chinese native sheep breed, has many advantages, including its reproductive performance, a high ratio of meat to bone, as well as tender and juicy meat, which customers often find appealing (Yue, 1996). However, the slow growth rates of Hu sheep have restricted the development and utilization of their excellent genetic resources. Thus, there is a need for more research to be conducted on the regulatory mechanisms underlying the muscle development of this local breed.

Mutton production is known to be closely tied to the muscle development of sheep. The process of mammalian skeletal muscle formation includes the proliferation, differentiation, and fusion of myogenic precursor cells into polynuclear muscle fibers (Buckingham, 2017). During the growth and regeneration of postpartum muscle fibers, the precursor cells to adult muscle that are located on the muscle fibers under the basal plate are called satellite cells (Mauro, 1961; Cossu and Biressi, 2005). These cells are responsible for the growth, hypertrophy, and regeneration of skeletal muscle after birth (Sun et al., 2017). When muscle is injured, activated satellite cells, which have been widely shown to be important adult stem cells, can proliferate and differentiate to form new muscle fibers (Beauchamp et al., 2000). Satellite cells of many animals have been successfully isolated, and they have become important materials for simulating the proliferation and differentiation of muscle cells *in vitro* (Nguyen et al., 2010). Therefore, Hu sheep skeletal muscle satellite cells (SMSCs) were used in this study to characterize the regulatory mechanism of genes related to muscle development and hypertrophy. An increasing number of genes regulating proliferation and differentiation have been found in SMSCs, such as Yes-associated protein 1 (*YAP1*) (Tremblay et al., 2014), *MyoG* (Jin et al., 2000), *MyoD* (Jin et al., 2000), *MyHC* (Chen et al., 2018), and *CSRP3* (Han et al., 2019).

YAP1 is one of the key central regulators of the Hippo signaling pathway, is widely expressed in mammalian tissues (Overholtzer et al., 2006), and functions in the proliferation of muscle satellite cells (Tremblay et al., 2014; Mohamed et al., 2016). YAP can positively regulate skeletal muscle size via interaction with TEAD transcription factors (Watt et al., 2015). Ser127 phosphorylation of YAP1 is essential for the terminal differentiation of mice myoblast (C2C12) cells (Watt et al., 2010). In a previous study, we successfully established a line of Hu sheep SMSCs and showed that YAP1 in the Hippo channel can regulate the proliferation and differentiation processes of the SMSCs by participating in the regulation of TGF-β/Smad pathway activity (Su, 2015).

Many studies have reported that microRNA (miRNA) can regulate gene expression by regulating post-transcriptional translation (Lu and Rothenberg, 2018). The endogenous miRNA precursor containing a stem-ring structure can be cut into mature miRNA by the Dicer enzyme during its transfer to cytoplasm (Bak and Mikkelsen, 2014). It combines with the miRNA response element contained in the 3′-untranslated regions (3′-UTR) of mRNA in a fully or partially complementary way to promote the degradation or inhibit the translation of targeted mRNA (Salmena et al., 2011). A large number of miRNAs participate in muscle growth and development, such as miR-206 (Vergara et al., 2018), miR-125b-5p (Qiu et al., 2019), miR-17, and miR-19 (Kong et al., 2019). For example, the disruption of miR-29 leads to abnormal differentiation of vascular smooth muscle cells (Cushing et al., 2015) and contributes to dystrophic muscle pathogenesis (Wang et al., 2012). However, the overexpression of miR-29 can also lead to congenital muscular dystrophy (Liu C. et al., 2019). These findings suggest that miR-29 may play different roles in different species and tissues.

Long noncoding RNA (lncRNA) is defined as noncoding RNA with lengths greater than 200 nt. It can play a role in muscle growth and morphogenesis and can be reproduced *in vitro* (Buckingham and Vincent, 2009). The rapid development of information technology and increasingly sophisticated research techniques have revealed that lncRNA plays an important role in regulating muscle growth and development in animals. For example, lncRNA can participate in skeletal muscle growth and development as competing endogenous RNA (ceRNA). lncRNA can regulate the balance of muscle by affecting corresponding miRNA, and its expression level has an important influence on several muscle diseases, such as myocardial infarction and Duchenne muscular dystrophy (Eisenberg et al., 2007). lncRNA-AK017368 was found to be highly expressed in skeletal muscle cells and can function as a ceRNA of miR-30c to promote the proliferation of myoblasts by weakening the function of miR-30c (Liang et al., 2018). lncIRS1, a sponge of the miR-15 family, was shown to regulate the expression of *IRS1* and promote skeletal muscle production (Li et al., 2019).

Research on muscle growth in Hu sheep, an important sheep breed native to China, is still in its incipient stages. Indeed, our understanding of the molecular mechanisms underlying the regulation of muscle growth, especially the regulation of lncRNA, is extremely limited. YAP1 plays an important role in skeletal muscle development; however, the underlying mechanism responsible for the beneficial effect of YAP1 in SMSCs remains unclear. This study aimed to explore the regulatory molecular pathway of lncRNA-miRNA-mRNA involved in mediating the function of YAP1. Our results will aid future studies of the breeding of Hu sheep as well as studies examining the molecular mechanisms underlying sheep muscle growth.

## Materials and Methods

### Ethics Statement

All experimental procedures were carried out in strict accordance with the guidelines for the care and use of laboratory animals in Jiangsu Province (License Number: 45) and the recommendations of the Animal Protection and Use Committee of the Ministry of Agriculture of China (License Number: 39). The protocol was approved by the Animal Care and Use Committee at Yangzhou University.

### Sample Collection

After intravenous injection of 2% lidocaine hydrochloride (4 mg/kg) to anesthetize sheep, 2 g skeletal muscle tissues were sampled (by slaughter) from *longissimus dorsi* of three 1-month-old and three 6-month-old Hu sheep and were immediately stored in liquid nitrogen. Sheep were fed in Suzhou Sheep Farm (Suzhou, Jiangsu, China). After intravenous injection of 2% lidocaine hydrochloride (240 mg, 4 mg/kg) to anesthetize ewes, a 90-day-old Hu sheep fetus was removed from the mother’s body by surgery to separate the SMSCs. Sheep were provided by Hua Mai Sheep Farm (Yangzhou, Jiangsu, China).

### Cell Acquisition and Culture

The 4th–5th generation SMSCs used in this study were isolated, identified, and cultured following a previously described method (Wu et al., 2012). Specifically, HEK293T cells kept in our lab and SMSCs were cultured in DMEM/F12 supplemented with 10% FBS and 1% penicillin-streptomycin (Gibco, Grand Island, NY, United States) at 37°C with 5% CO_2_. When the cells reached the logarithmic growth stage, they were seeded in different culture plates before transfection.

### Plasmid Construction

All the primer sequences used in plasmid construction are listed in Table 1.

**TABLE 1:** Primers information for plasmid construction.

Name	Primer sequence (5′ to 3′; F: forward, R: reverse)	Product size (bp)
lncRNA-overexpression	F (*HindIII*): AGCTGGCTAGCGTTTAAACTT**aagctt**GTCTCTGGAGCGGGCGC	762
	R (*BamHIII*): AGAATTCCACCACACTGGACTAGT**ggatcc**CACTCTTCATCTCAACTCCCAGC	
YAP1-wild	F (*HindI*): CAATGAAAAGATCCTTTATT**aagctt**GCCTACAATTTGCCATTAAGCC	535
	R (*MluI*): GAGCTCATAGGCCGGCATAG**acgcgt**AATGCCACCCAATACAACCA	
YAP1-mutant	F: CAGCACCGTG**tg**G**at**TGAAGGACATGGTG R: CACCATGTCCTTCA**at**C**ca**CACGGTGCTG	6,927
lncRNA-wild	F (*HindIII*): CAATGAAAAGATCCTTTATT**aagctt**GTCTCTGGAGCGGGCGC	763
	R (*MluI*): GAGCTCATAGGCCGGCATAG**acgcgt**CACTCTTCATCTCAACTCCCAGC	
lncRNA-mutant 1	F: GGAGGGTCTGGG**attct**CTCAGATTCAGG R: CCTGAATCTGAG**agaat**CCCAGACCCTCC	7,186
lncRNA-mutant 2	F: GGAGGGTCTGAGT**aactt**TGAGGTTCAGGT R: TCCTGAACCTCA**aagtt**ACTCAGACCCTCC	7,186
lncRNA-mutant 3	F: AGGAGGGACCGGG**aatct**CTCAGGTTCAGGAA R: TTCCTGAACCTGAG**agatt**CCCGGTCCCTCCT	7,186

*In wild and overexpression types, the nucleotides in lowercase indicate the restriction enzyme sites. But, in mutant types, the nucleotides in lowercase indicate the mutant site.*

To overexpress lncRNA CTTN-IT1, the primers, which contained *Hind III* and *BamH I* restriction sites and vector homologous fragments, were used to amplify the full-length sequence of CTTN-IT1 (sequence can be found in Supp CTTN-IT1 Sequence). Next, the “lncRNA-overexpression plasmid” was constructed using the full-length sequence of CTTN-IT1 and the pcDNA3.1(+) vector (ThermoFisher, Shanghai, China) digested by *Hind III* and *BamH I* restriction enzymes (Takara, Kusatsu, Shiga, Japan).

For the dual-luciferase assay, wild and mutant 3′-UTRs (containing the miR-29a binding site) of *YAP1* were amplified and linked downstream of the luciferase gene of the pmiRNA-REPORT Luciferase vectors (ThermoFisher, Shanghai, China) digested by *Hind III* and *Mlu I* restriction enzymes (Takara, Kusatsu, Shiga, Japan). The wild 3′-UTR of *YAP1* was used as a template to construct the recombinant dual-luciferase plasmid of the “YAP1-wild” type and was copied by PCR with designed primers, which contained vector fragments and restriction endonuclease sites. Meanwhile the “YAP1-mutant” plasmid was modified from the “YAP1-wild” plasmid with a Fast Site-Directed Mutagenesis Kit (Tiangen, Beijing, China) per the manufacturer’s protocol.

The full-length sequence of CTTN-IT1 was amplified by PCR with primers containing *Hind III* and *Mlu I* restriction sites and vector homologous fragments to construct the recombinant dual-luciferase plasmid of the “lncRNA-wild” type. To explore the actual effects of the three potential binding sites predicted by software using the “lncRNA-wild” plasmid as a template, the base pairs involved in the miR-29a binding were modified using a Fast Site-Directed Mutagenesis Kit; and the plasmid was named the “lncRNA-mutant 1/2/3” plasmid. The sequences of wild and mutated fragments were shown in Supplementary Material 2.

Before constructing the plasmid, all of the DNA fragments were purified by gel cutting with a Universal DNA Purification Kit (Tiangen, Beijing, China). Next, the target segments were linked with the vector by a Trelief SoSoo Cloning Kit (Tsingke, Beijing, China) per the manufacturer’s protocol.

### Cell Transfection

SMSCs at the exponential phase were digested by trypsin and seeded in 6, 24, or 96-well plates at a concentration of 3 × 10^4^ cells/cm^2^. When cells were approximately 80% confluent, 100 nM of miRNA mimic/miRNA mimic-NC or 200 nM of miRNA inhibitor/miRNA inhibitor-NC and 50 nM of siRNA (All from RiboBio, Guangzhou, China) were transfected into the cells using Lipofectamine RNAiMAX Reagent (ThermoFisher, Shanghai, China) per the manufacturer’s protocol. Next, pcDNA3.1(+) vector was used as a Blank, and the lncRNA-overexpression plasmid was transfected or co-transfected with miRNA-mimics or other substances using jetPRIME (Polyplus-transfection, New York, NY, United States) per the manufacturer’s protocol.

siRNAs that can inhibit CTTN-IT1 were designed and produced by RiboBio Co. Ltd. (Guangzhou, China). To regulate the content of miRNA in cells, the miRNA mimic and mimic-NC as well as the miRNA inhibitor and inhibitor-NC, which were used for transfections into SMSCs or HEK293T cells, were synthesized by RiboBio Co. Ltd. as well.

### EdU Staining Assay

A total of 1 × 10^4^ cells/well of SMSCs were seeded in 96-well plates with five replicates per condition. When cells reached 60% confluence, 3 μg/mL of the CTTN-IT1 overexpression vector or pcDNA3.1(+) vector and 100 nM of miR-29a mimic, mimic-NC, or 200 nM miR-29a inhibitor were co-transfected into SMSCs. After 12 h, cells were treated with EdU reagent, which was contained in a Cell-Light EdU Apollo567 In Vitro Kit (RiboBio, Guangzhou, China), for 2 h and stained per the manufacturer’s instructions. Three groups of photos (400× magnification) per well of the stained cells were taken at random under a fluorescence microscope (Nikon, Tokyo, Japan). The ratio of EdU positive cells (Apollo567 staining cells/Hoechst staining cells) was then calculated.

### RNA Isolation and RT-qPCR

To extract RNA, muscle tissue was ground into powder in liquid nitrogen, and the SMSCs were cultured for 48 h after transfection. Several tests were then conducted, and the steps for each are described below.

To detect the expression profile of miRNA, the miRNA of muscle tissue or cells was extracted using an miRcute miRNA Isolation Kit (Tiangen, Beijing, China) per the manufacturer’s instructions. The miRNA was reverse-transcribed into cDNAs by modifying the miRNA with a poly (A) tail using an miRcute Plus miRNA First-Strand cDNA Synthesis Kit (Tiangen, Beijing, China) per method A of the manufacturer’s instructions.

To measure the expression profiles of mRNA and lncRNA, the total RNA of the SMSCs was isolated using an RNAsimple Total RNA Kit. The cDNA was then synthesized using an RNA First-Strand cDNA Synthesis Kit (Tiangen, Beijing, China).

An Applied Biosystems 7900 Fast Real-Time PCR system (ThermoFisher, Shanghai, China) was used to conduct RT-qPCR. Primer information is shown in Table 2.

**TABLE 2:** Primer information for RT-Qpcr.

Target	Accession number	Primer sequence (5′-3′; F: forward, R: reverse)	Product size (bp)
miRNA-200	NR_107956.1	F: TGGTAACGATGTCGTATCCAGT	Variable
		R: sequence provided by kit	
miRNA-181a	NR_107883.1	F: GCTGTCGGTGAGTGTCGTAT	Variable
		R: sequence provided by kit	
miRNA-541-3p	NR_107928.1	F: AATCCGGCCTCTGTCGTATC	Variable
		R: sequence provided by kit	
miR-29a	NR_107880.1	F: GCACCATCTGAAATCGGTTGT	Variable
		R: sequence provided by kit	
YAP1	XM_015100720.2	F: GACAGCGGACTGAGCATGAG	108
		R: CAGGGTGCTTTGGTTGATAGTG	
MyHC	XM_004010325.3	F: TCGTCAAGGCCACAATTTG	101
		R: CTGCTGCAACACCTGGTCCT	
MyoD	NM_001009390.1	F: GCTCCAGAACCGCAGTAAGTT	106
		R: CGGCGACAGCAGCTCCATA	
MyoG	NM_001174109.1	F: AATGAAGCCTTCGAGGCCC	101
		R: CGCTCTATGTACTGGATGGCG	
lncRNA CTTN-IT1	Not in the database	F: TGACATGGCTGCTGTGACTG	189
		R: TTACACGCAGACAGGGTCATC	
U6	XM_012096728.2	F: CTCGCTTCGGCAGCACA	95
		R: AACGCTTCACGAATTTGCGT	
GAPDH	NM_001190390.1	F: TCTCAAGGGCATTCTAGGCTAC	151
		R: GCCGAATTCATTGTCGTACCAG	

The RT-qPCR reaction solution system for miRNA using an miRcute Plus miRNA qPCR Kit (Tiangen, Beijing, China) is shown in Supplementary Table S1. The RT-qPCR program for miRNA was as follows: 95°C for 15 min, followed by 40 cycles of 94°C for 20 s and 60°C for 34 s.

The RT-qPCR reaction solution system for mRNA and lncRNA using SYBR Premix Ex Taq II (TaKaRa, Dalian, China) is shown in Supplementary Table S2. The RT-qPCR program for mRNA and lncRNA, which was the same as the method provided by the guide, was as follows: 95°C for 30 s, followed by 40 cycles of 95°C for 5 s and 60°C for 34 s and, finally, 95°C for 15 s and 60°C for 60 s.

There were three biological replicates for each condition. The results were analyzed using the 2^–ΔΔ*CT*^ method (Livak and Schmittgen, 2001) with either *GAPDH* or *U6* as the internal reference genes.

### Dual-Luciferase Assay

To elucidate the relationship between miR-29a and *YAP1* and between miR-29a and CTTN-IT1, HEK293T cells were seeded in a 24-well plate and co-transfected with 500 ng/mL YAP1-wild, YAP1-mutant, lncRNA-wild, lncRNA-mutant, pRT-TK (RiboBio, Guangzhou, China) plasmid, and 100 nM of miR-29a mimic (and mimic-NC) or 200 nM of miR-29a inhibitor (and inhibitor-NC). The FuGENE HD Transfection Reagent (Promega, Fitchburg, WI, United States) was used for the transfection per the manufacturer’s protocol. The ratio of the transfection reagent to plasmid was 3:1. Forty-eight hours later, the dual-luciferase assay was conducted with a Promega Dual-Glo Luciferase-Assay System (Promega, Fitchburg, WI, United States) per the manufacturer’s protocol. The main steps were as follows:

1. The SMSCs from different treatments were first washed twice with PBS, 100 μL/well of PLB lysate was added, and the cells were fully lysed by shaking at room temperature for 15 min.
2. Twenty microliters of cell lysate was added into the 96-well enzyme plate, and 100 μL of Luciferase Reagent was added to test the luciferase activity of fireflies.
3. One hundred microliters of Stop & Glo Reagent was added into the well to detect the luciferase activity of *Renilla*.
4. Luciferase detection was run at the setting of 1–2 s delay and 5–10 s reading. The data were presented as relative luciferase activity (Firefly/*Renilla*, OD/OD) and were normalized by a control well. Each group consisted of three replicates.

### Western Blot

The protein samples were extracted from SMSCs after the miR-29a mimic (and mimic-NC) or miR-29a inhibitor (and inhibitor-NC) and lncRNA-overexpression plasmid [and pcDNA3.1(+)] were co-transfected into the cells for 48 h. The concentration of protein samples was measured and standardized before the samples were compressed by 5% spacer gel (80 V, 20 min) and separated by 10% separation gel (100 V, 80 min) through electrophoresis. Next, the separated proteins were transferred from gel to a polyvinylidene difluoride (PVDF) membrane by electricity transfer (200 mA, 1 h). After being blocked with 5% skimmed milk for 1 h at room temperature, the PVDF membranes were kept in primary antibodies [against YAP1(ab205270, 1:1000), MyoG(ab187506, 1:1000), MyoD(ab126726, 1:1000), MyHC(ab91506, 1 μg/ml), and β-actin(ab6276, 1:5000)] (Abcam, Cambridge, United Kingdom) overnight at 4°C and then incubated with the HRP-conjugated secondary antibody goat-anti-mouse IgG (Zhongshan-Bio, Beijing, China) at room temperature for 2 h. The PVDF membranes were washed with PBST several times, 5 min per wash, after incubation in antibodies. Finally, the developer was added to the PVDF membrane and was placed at room temperature for 1 min. The membrane was then wrapped with plastic wrap (bubbles were avoided as much as possible) in a dark room, and the membrane was quickly pasted on the X-ray film for exposure. The image was developed and washed in the film-developing machine. The exposure time was adjusted until the best strip appeared.

### Software Prediction

To predict the potential target-related miRNAs in the 3′-UTR regions of *YAP1* of Hu sheep, the potential miRNAs were selected by two online softwares: miRBase^1^ and miRanda^2^. Findtar3^3^ and RNA22^4^ were used to predict the existence of lncRNA that were potentially targeted by miR-29a.

### Statistical Analysis

Excel 2013 (Microsoft, Redmond, WA, United States) was used for data analyses. SPSS 16.0 (IBM, Armonk, NY, United States) was used to calculate the significance of *t*-tests. In addition to using multiple comparisons (LSD) in Figure 4D, the significance for other figures was calculated using independent sample *t*-tests. All of the bar charts were drawn with GraphPad Prism 6 (GraphPad Software, San Diego, CA, United States). The marker “^∗^” represented a significant difference between the experimental group and the control group (*P* < 0.05), and “^∗∗^” represented an extremely significant difference between the experimental group and the control group (*P* < 0.01).

## Results

### miR-29a Regulates YAP1 Expression by Targeting Its 3′-UTR

To explore the miRNAs that target *YAP1*, online programs, such as Findtar3, miRBase, RNA22, and miRanda, were used to predict the miRNAs that could potentially target the 3′-UTR of *YAP1* in sheep. Four miRNAs were found to potentially target *YAP1* gene by binding to the 3′-UTR of *YAP1* (Supplementary Table S3). Among these four miRNAs, miR-29a has already been shown to play an important role in skeletal muscle development of mice in several papers (Galimov et al., 2016; Zhou et al., 2016; Muluhngwi et al., 2017), consequently, the relationship between miR-29a and *YAP1* was studied further.

**FIGURE 1:**
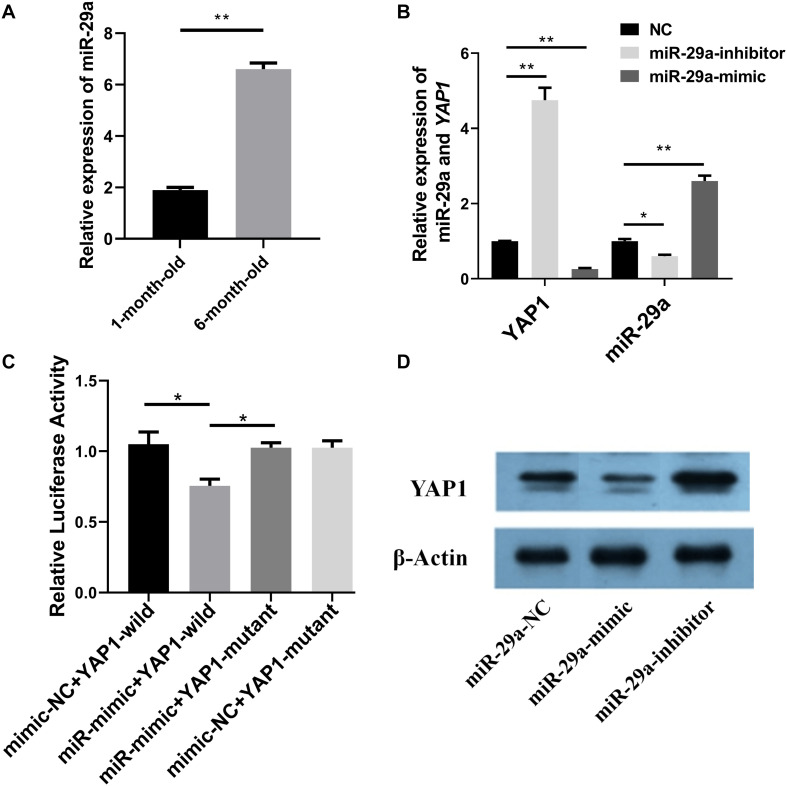
Interaction between miR-29a and *YAP1*. **(A)** The expression of miR-29a in the *longissimus dorsi* muscle between 1-month-old and 6-month-old Hu sheep. **(B)** The relative expression level of miR-29a and *YAP1* after miR-29a mimic or inhibitor was transfected into SMSCs. **(C)** Results of the dual-luciferase reporter assay to confirm the potential interaction between miR-29a and *YAP1*, after YAP1-wild or YAP1-mutant, recombined dual-luciferase reporter plasmid, and miR-29a mimic (and mimic-NC) or miR-29a inhibitor (and inhibitor-NC) were co-transfected into HEK293T cells. **(D)** The expression of YAP1 and β-actin was detected by western blot after miR-29a mimic and inhibitor were transfected into SMSCs. *P < 0.05 or **P < 0.01.

Our previous study showed that the expression of *YAP1* in slow-growing, 6-month-old Hu sheep was significantly lower than that in fast-growing, 1-month-old Hu sheep (Gao et al., 2016). RT-qPCR was then used to detect the expression of miR-29a in the *longissimus dorsi* muscle of 1-month-old and 6-month-old Hu sheep (Figure 1A). The relative expression level of miR-29a in the 6-month-old *longissimus dorsi* of Hu sheep was extremely significantly higher than those observed in 1-month-old *longissimus dorsi* (*P* < 0.01). The expression of miR-29a was negatively correlated with the expression of *YAP1*, indicating that *YAP1* was a potential target gene of miR-29a.

The effectiveness of the miR-29a mimic and inhibitor was detected by RT-qPCR after transfection of miR-29a mimic/mimic-NC and inhibitor/inhibitor NC into SMSCs (Figure 1B, right part). Transfection of both mimic and inhibitor was significantly different compared with the control (*P* < 0.05). Therefore, miR-29a mimics and inhibitors were used in subsequent experiments. The expression of *YAP1* was also detected by RT-qPCR after transfection of miR-29a mimic/mimic-NC and inhibitor/inhibitor NC into SMSCs (Figure 1B, left part). Transfection of the miR-29a mimic resulted in a highly significant decrease in the expression of *YAP1* (*P* < 0.01), and miR-29a inhibitor resulted in a highly significant increase in the expression of *YAP1* (*P* < 0.01). Thus, miR-29a can negatively regulate *YAP1*.

The dual-luciferase assay and western blot were used to verify the relationship between *YAP1* and miR-29a. YAP1-wild or YAP1-mutant was recombined with the dual-luciferase reporter plasmid, and miR-29a mimic/mimic-NC or miR-29a inhibitor/inhibitor-NC was co-transfected into HEK293T cells. After 48 h, the dual-luciferase reporter gene experiment was conducted. The relative activity of luciferase in the co-transfected miR-29a mimic and wild-type vector group was significantly lower than that in the control group (*P* < 0.05), while the relative luciferase activity in the co-transfected miR-29a mimic and mutated vector group was not significantly different from that in the control group (*P* > 0.05) (Figure 1C). Thus, the miR-29a seed region can bind to the target site of *YAP1*, consistent with our prediction. The western blot (Figure 1D) revealed that the miR-29a mimic could inhibit the expression of YAP1 protein and that the miR-29a inhibitor could increase its expression. Thus, miR-29a can reduce the expression level of YAP1 protein, suggesting that *YAP1* is the target gene of miR-29a.

### miR-29a Can Inhibit the Proliferation and Differentiation of SMSCs

**FIGURE 2:**
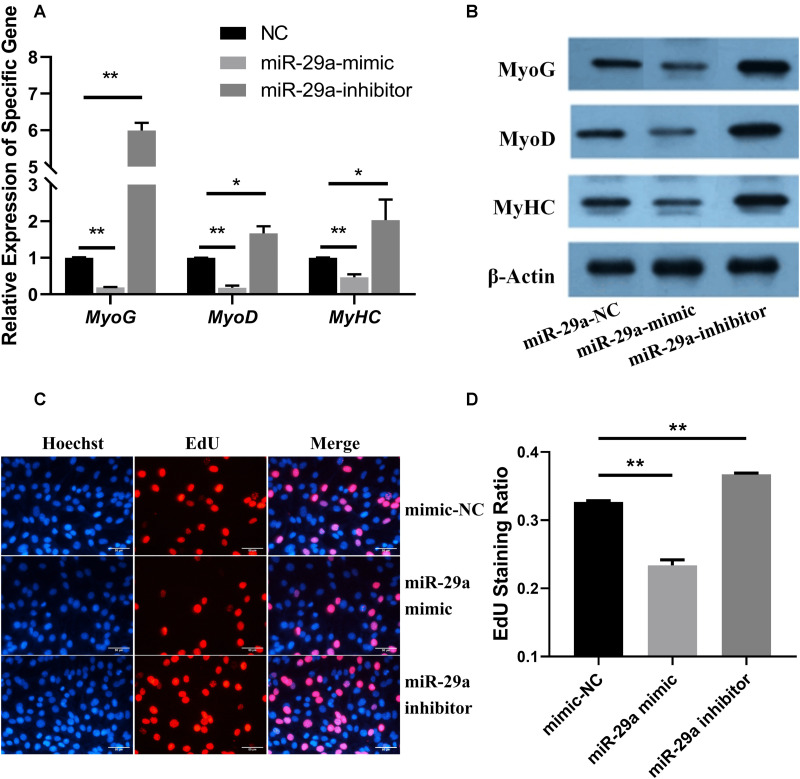
Functional analysis of miR-29a during the proliferation and differentiation of SMSCs. **(A)** The relative expression levels of *MyoG*, *MyoD*, and *MyHC* after the transfection of miR-29a mimic and inhibitor. **(B)** The protein expression of MyoG, MyoD, MyHC, and β-actin was detected by western blot after miR-29a mimic and inhibitor were transfected into SMSCs. **(C)** Images of the results of the EdU staining assay after miR-29a mimics, mimic-NC, and inhibitors were transfected into SMSCs. The short white lines in each picture represent 50 μm. **(D)** Data analysis of the EdU results. Higher values represent more rapid cell proliferation. *P < 0.05 or **P < 0.01.

YAP1 can regulate the proliferation and differentiation processes of the SMSCs. To determine whether miR-29a can regulate the proliferation and differentiation of SMSCs by regulating *YAP1*, the mRNA and protein expression of the marker genes of cell differentiation including MyoG, MyoD, and MyHC (Figures 2A,B) were detected after miR-29a mimic and inhibitor were transfected into SMSCs, and the cell proliferation ability was detected by the EdU staining assay (Figures 2C,D).

The relative expression levels of *MyoG*, *MyoD*, and *MyHC* were significantly lower in the control group (*P* < 0.01) after overexpression of miR-29a by transfection of the miR-29a mimic, but they were significantly higher than the control group after inhibiting endogenous miR-29a (*P* < 0.05; Figure 2A). Furthermore, western blot was used to detect the protein levels of these genes related to muscle differentiation after transfection of miR-29a mimic or inhibitor (Figure 2B). After overexpression of miR-29a in SMSCs, the protein expression levels of these genes were lower than those in the negative control group; in contrast, after inhibition of miR-29a, the protein expression levels of these genes in muscle were higher than those in the negative control group. These results thus suggested that miR-29a might inhibit the differentiation of SMSCs.

After miR-29a mimics, mimic-NC, and inhibitors were transfected into SMSCs, the proliferation of Hu sheep SMSCs was detected by the EdU staining assay (Figures 2C,D). Compared with the control group, the transfection of miR-29a mimic could significantly reduce cell proliferation, while the transfection of miR-29a-inhibitor could significantly increase cell proliferation (*P* < 0.05). These results indicated that miR-29a could inhibit the proliferation of Hu sheep SMSCs by silencing *YAP1* mRNA expression.

### lncRNA CTTN-IT1 Upregulates YAP1 by Acting as a ceRNA Through the Absorption of miR-29a

Two online programs, RNA22 and Findtar3, were used to predict the potential lncRNA-containing target sites binding to miR-29a. Eventually, the CTTN-IT1 and its three binding sites potentially targeted by miR-29a were identified (Table 3).

**TABLE 3:** Target prediction of CTTN-IT1 and oar-miR-29a.

LncRNA	Binding site No.	Position	Total score	Total energy
CTTN-IT1	1	399-420	600	−73.45
	2	529-550	611	−77.12
	3	629-650	931	−118.92

**FIGURE 3:**
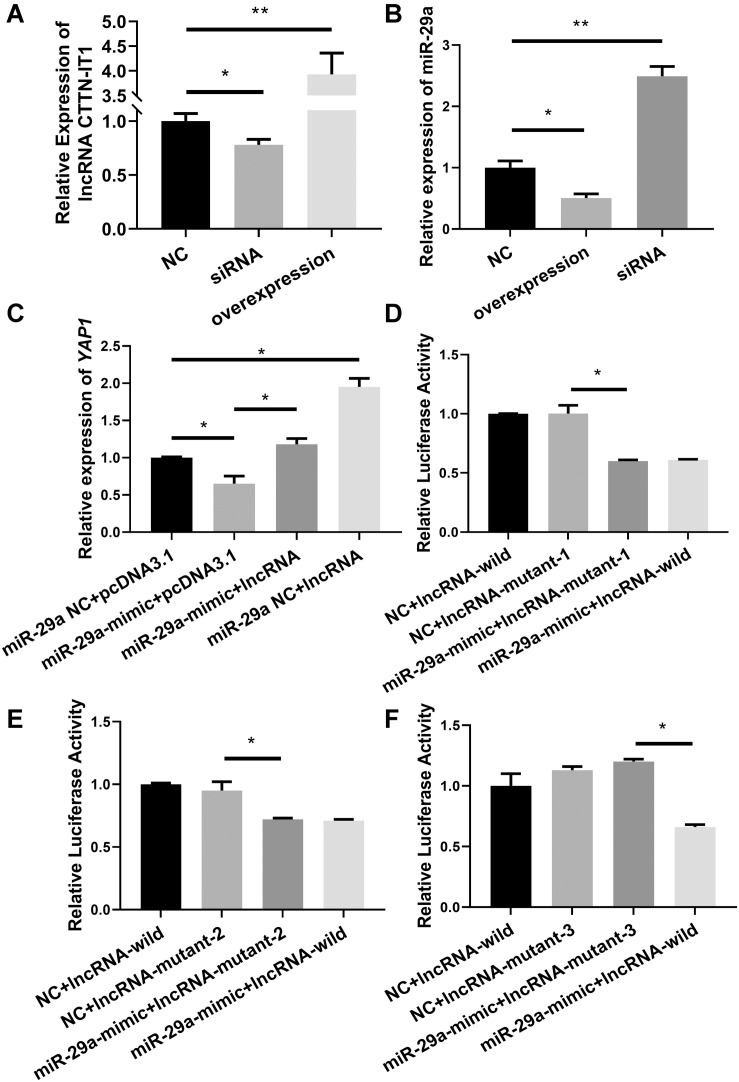
Verification of target regulation relationship between miR-29a and CTTN-IT1. **(A)** The relative expression level of CTTN-IT1 after overexpression or interference of CTTN-IT1 in SMSCs. **(B)** The relative expression level of miR-29a after overexpression or interference of CTTN-IT1 in SMSCs. **(C)** The relative expression level of *YAP1* after lncRNA-overexpression plasmid [pcDNA3.1(+) used as Blank] was co-transfected with miR-29a mimic or miR-29a mimic-NC into SMSCs. **(D–F)** These three figures are the results of dual-luciferase reporter assay. Relative luciferase activity was assessed after co-transfecting the miR-29a mimic (miR-29a mimic-NC used as Blank) with the wild or the mutant plasmids of CTTN-IT1 into HEK293T cells. Mutant plasmids of three different binding sites listed in Table 3 were used in figures **(D–F)**, respectively. The “lncRNA” in **C** means the transfection of lncRNA-overexpression plasmid. *P < 0.05 or **P < 0.01.

First, the validity of the plasmid used for transfection was tested. The relative expression level of CTTN-IT1 was detected by RT-qPCR after the lncRNA-overexpression plasmid, lncRNA-siRNA, or its negative control were transfected into Hu sheep SMSCs. The relative expression level of CTTN-IT1 in the overexpression group was significantly higher than that in the negative control group (*P* < 0.01), and its expression level in the interference group was significantly lower than that in the negative control group (*P* < 0.05) (Figure 3A). Thus, the lncRNA-overexpression plasmid was constructed successfully, and siRNA was effective enough for use in the subsequent experiment.

To verify the effect of CTTN-IT1 on miR-29a, the relative expression level of miR-29a was detected by RT-qPCR after the lncRNA-overexpression plasmid, lncRNA-siRNA, or its negative control were transfected into Hu sheep SMSCs. Compared with the negative control group, the relative expression level of miR-29a in the CTTN-IT1 overexpression group was significantly lower (*P* < 0.05). Transfection of lncRNA-siRNA showed that the relative expression level of miR-29a in the lncRNA-siRNA group was significantly higher than that in the negative control group (*P* < 0.01) (Figure 3B). Thus, CTTN-IT1 could effectively reduce mature miR-29a in SMSCs.

To characterize the effect of CTTN-IT1 on *YAP1* through its reduction of miR-29a, lncRNA-overexpression plasmid, or pcDNA3.1 were co-transfected with miR-29a mimic or miR-29a mimic-NC into Hu sheep SMSCs. The co-transfected pcDNA3.1, and miR-29a mimic-NC was the control group. RT-qPCR was used to detect the relative expression level of *YAP1* (Figure 3C). Co-transfection of the miR-29a mimic and pcDNA3.1 could significantly reduce *YAP1* content in cells (*P* < 0.05). However, overexpression of miR-29a and CTTN-IT1 significantly increased the expression of *YAP1* (*P* < 0.05) relative to the overexpression of miR-29a alone. In general, overexpression of CTTN-IT1 could significantly increase the expression of *YAP1* (*P* < 0.05). Overall, CTTN-IT1 can positively regulate *YAP1* expression, and overexpression of CTTN-IT1 can restore the expression of *YAP1* from inhibition by miR-29a.

To identify the key binding site of CTTN-IT1 during this regulatory process, different plasmids (lncRNA-wild/lncRNA-mutant) were co-transfected with miR-29a mimic/mimic-NC into HEK293T cells. The co-transfected lncRNA-wild, and miR-29a mimic-NC was the control group. After 48 h, the dual-luciferase assay revealed that the activity of luciferase in the co-transfected miR-29a mimic and the lncRNA-wild group in all three histograms was significantly lower than that in the control group (*P* < 0.05). Compared with binding sites 1 and 2, only binding site 3 showed no significant difference in relative luciferase activity between the co-transfected miR-29a mimic and lncRNA-mutant group compared with the control group (*P* > 0.05) (Figures 3D–F). Thus, binding site 3 (located from 629 to 650 bp on CTTN-IT1) was the binding site of miR-29a and CTTN-IT1.

### lncRNA CTTN-IT1 Affects the Differentiation and Proliferation of SMSCs

**FIGURE 4:**
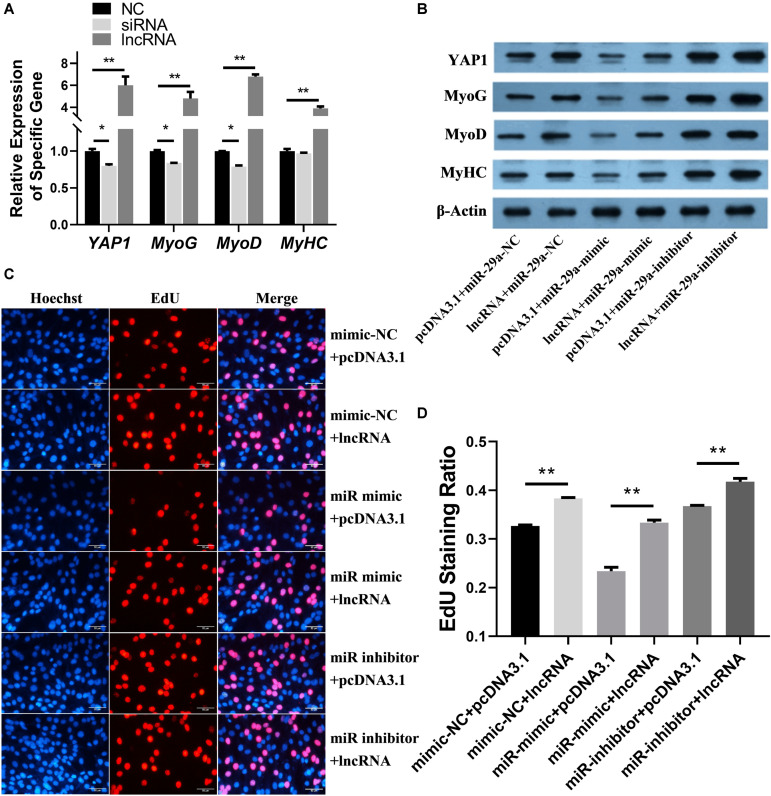
Functional analysis of miR-29a and CTTN-IT1 during the proliferation and differentiation of SMSCs. **(A)** The relative expression levels of *YAP1*, *MyoG*, *MyoD* and *MyHC* after lncRNA-overexpression or LncRNA-siRNA were transfected into SMSCs [pcDNA3.1(+) used as NC]. **(B)** The protein expression of YAP1, MyoG, MyoD, MyHC and β-actin was assessed by western blot after the transfection of lncRNA-overexpression plasmid [pcDNA3.1(+) used as Blank], miR-29a mimic or miR-29a inhibitor (miR-29a mimic-NC used as Blank) in SMSCs. **(C)** Images of the results of the EdU staining assay after miR-29a mimics, mimic-NC and inhibitors were co-transfected with LncRNA-overexpression plasmid into SMSCs. The short white lines in each picture represent 50 μm. **(D)** Data analysis of the EdU results. Higher values represent faster cell proliferation. All the “lncRNA” this figure means the transfection of lncRNA-overexpression plasmid. *P < 0.05 or **P < 0.01.

The mRNA expression levels of *YAP1*, *MyoG*, *MyoD*, and *MyHC* (Figure 4A) were assessed after lncRNA overexpression or lncRNA-siRNA [pcDNA3.1(+) as the control group] were transfected into SMSCs. Overexpression of CTTN-IT1 significantly increased the expression level of *YAP1* (*P* < 0.01), and the interference of CTTN-IT1 significantly decreased the expression level of *YAP1* (*P* < 0.05). Thus, CTTN-IT1 can regulate the expression of *YAP1*.

The relative expression levels of *MyoG*, *MyoD*, and *MyHC* were significantly higher than those of the control group (*P* < 0.01) after overexpression of CTTN-IT1. The transfection of siRNA could significantly down-regulate the relative expression levels of *MyoG* and *MyoD* (*P* < 0.05). However, siRNA transfection had no significant effect on the expression of *MyHC* (*P* > 0.05). Thus, CTTN-IT1 can promote the expression of genes related to the differentiation of SMSCs.

After lncRNA-overexpression plasmid [pcDNA3.1(+) was used as a blank] and miR-29a mimic or miR-29a inhibitor (miR-29a mimic-NC used as Blank) were co-transfected into SMSCs, the proteins expression of YAP1, MyoG, MyoD, MyHC, and β-actin was detected by western blot (Figure 4B). The protein expression level in the CTTN-IT1 overexpressed group (lncRNA+miR-29a-NC) was higher than that in the negative control group (pcDNA3.1+miR-29a-NC). CTTN-IT1 can restore the protein expression of YAP1, MyoG, MyoD, and MyHC from inhibition by miR-29a in the lncRNA+miR-29a mimic group relative to the pcDNA3.1+miR-29a mimic group and pcDNA3.1+miR-29a-NC groups. The co-transfection of lncRNA-overexpression plasmid and miR-29a inhibitor had the highest protein expression levels. Thus, CTTN-IT1 can regulate the expression of YAP1 at the protein level and can increase the expression levels of MyoG, MyoD, and MyHC, proteins related to the differentiation of SMSCs.

After lncRNA-overexpression plasmid [pcDNA3.1(+) used as Blank] and miR-29a mimic or miR-29a inhibitor (miR-29a mimic-NC used as a blank) were co-transfected into SMSCs, the cell proliferation ability was assessed by the EdU staining assay (Figures 4C,D). Our results showed that overexpression of CTTN-IT1 could significantly increase the proliferation of SMSCs in each comparison (*P* < 0.01). Thus, CTTN-IT1 can promote SMSC proliferation, which was probably achieved by reducing the content of miR-29a to restore the function of *YAP1*.

In sum, miR-29a can inhibit the proliferation and differentiation of Hu sheep SMSCs by silencing *YAP1* and indirectly reducing the expression of *MyoG*, *MyoD*, and *MyHC*. CTTN-IT1 can promote the proliferation and differentiation of SMSCs by reducing the content of endogenous miR-29a.

## Discussion

YAP/TAZ act downstream in the Hippo pathway and have been extensively studied given their important functions (Piccolo et al., 2014). YAP1 can regulate cell proliferation, migration, and apoptosis (Lapi et al., 2008; Wang et al., 2009). Knockdown of YAP1 can decreased the viability of vascular smooth muscle cells, and overexpression of YAP1-5SA, which lacks five serine phosphorylation sites, found that YAP1 can inhibit the apoptosis induced by endoplasmic reticulum stress (Takaguri et al., 2017). Overexpression of YAP1 can lead to the hypertrophy of cardiac muscle cells (Del Re et al., 2013). Our previous study also showed that YAP1 is closely related to the proliferation and differentiation of Hu sheep muscle (Su, 2015). Research on YAP1 regulation has gradually deepened yearly. The expression of YAP1 protein can been down regulated by tunicamycin in vascular smooth muscle cells (Takaguri et al., 2017). The mRNA expression of *YAP1* can be targeted and reduced by some miRNAs such as miR-375 (Zhao et al., 2020) and miR-9 (Zheng et al., 2019) in cell line models of human diseases. However, the regulatory molecular mechanism of miRNA on the proliferation and differentiation of Hu sheep muscle through YAP1 remains unclear.

MiRNAs, miR-29a included, were involved in a variety of physiological processes like differentiation, disease, and development. The miR-29a not only participates in the formation of omental neovascularization (Han et al., 2018), diabetes (Zhang et al., 2017; Huang et al., 2019), and tumors (Liu et al., 2018) but also in the growth and development of skeletal muscle (Galimov et al., 2016; Zhou et al., 2016; Muluhngwi et al., 2017). The growth rate of Hu sheep peaks at the age of 1 month and gradually tends to flatten at the age of 6 months (Chen et al., 2014). This difference in growth rate allowed us to compare the differences in the expression of miRNAs between 1- and 6-month-old sheep and to identify the miRNAs associated with *YAP1*. The relative expression level of miR-29a was significantly higher at the age of 6 months than at the age of 1 month, indicating that the expression of miR-29a may increase as the proliferation rate of Hu sheep muscle cells decreases. We therefore speculated that miR-29a plays a negative role in the proliferation of Hu sheep satellite cells. The expression profile of miR-29a in skeletal muscle documented in fetal and mature goats (Wang et al., 2014) and pigs (Siengdee et al., 2015) was similar to the profile observed in our study. Then we found that miR-29a can inhibit the proliferation and differentiation of SMSCs in Hu Sheep by targeting the 3′-UTR of *YAP1*.

Furthermore, RT-qPCR, EdU, and western blot technology were used to reveal the function of miR-29a. Considering the crucial roles of *MyoD*, *MyoG*, and *MyHC* in the differentiation of muscle satellite cells (Tedesco et al., 2010), these genes were selected as the marker genes of myocyte differentiation in this study. Both RT-qPCR and western blot revealed that miR-29a may play a role in inhibiting SMSC differentiation. Previous studies have also suggested that *YAP1* can facilitate the proliferation of SMSCs (Watt et al., 2010; Liu et al., 2017) but inhibit their differentiation (Sun et al., 2017). Previous studies have also suggested that miR-29a can inhibit the growth of skeletal muscle (Wang et al., 2011; Liu C. et al., 2019) and the proliferation of satellite cells and promote the differentiation of satellite cells (Kapinas et al., 2010; Wei et al., 2013). Our results also showed that miR-29a can inhibit the proliferation of SMSCs, which is consistent with previous studies. However, our preliminary results showed that miR-29a can inhibit the genes related to SMSC differentiation, indicating that miR-29a might inhibit both proliferation and differentiation in Hu sheep SMSCs. One potential explanation is that the functions of miR-29a differ between species. In addition, several studies have revealed similar expression profiles between *MyoG* (Lv et al., 2015), *MyHC* (Gao et al., 2016), and *YAP1* (decreases with age), indicating that miR-29a may inhibit both *YAP1* and genes related to differentiation in Hu sheep SMSCs. Another potential explanation is that, in addition to *YAP1*, there might be other genes targeted by miR-29a involved in the proliferation and differentiation of SMSCs. All of these possibilities require further study.

LncRNA CTTN-IT1, is located within the intron between exon 10–11 of *CTTN* on chromosome 21 (49352559 bp to 49353268 bp) of sheep. Some researchers believe that lncRNAs regulate cell functions through competitively binding miRNAs (Geng and Tan, 2016; Marchese et al., 2017). In line with this idea, after verifying that CTTN-IT1 can regulate the expression of miR-29a, a dual-luciferase vector and siRNA were designed for three potential miR-29a target sites of CTTN-IT1 to complete a series of experiments. CTTN-IT1 was found to play a positive role in *YAP1* regulation through the third binding site by reducing the natural content of miR-29a. It means that CTTN-IT1 can act as a ceRNA and function as a sponge that absorbs miR-29a. Thus, overexpression of CTTN-IT1 can up-regulate the expression of *YAP1* and genes related to muscle differentiation, and when the miR-29a mimic was transfected, the opposite pattern was observed. The result of the EdU staining assay also verified that CTTN-IT1 can promote the proliferation of SMSCs. CTTN-IT1 in this experiment has the same effect in promoting the proliferation of muscle cells as ANRIL (Tan et al., 2019), lncRNA-MEG3 (Bai et al., 2019), and Linc00299 (Liu Y. et al., 2019). There is another lncRNA has been reported can also regulate *YAP1*. LncRNA PFAR can positive regulate *YAP1* by acts as a ceRNA of miR-138 to promote lung fibroblast activation and fibrosis (Zhao et al., 2018). Other lncRNAs have been reported to be involved in the differentiation or proliferation of SMSC. LncR-125b can promotes the differentiation of goat SMSC by absorbing miR-125b (Zhan et al., 2019). Lnc133b can regulate bovine SMSC proliferation and differentiation by mediating miR-133b (Jin et al., 2017).

In sum, the results of the present work identify a lncRNA CTTN-IT1 witch can function as a ceRNA to regulate *YAP1* by competitively binding to miR-29a. In addition, CTTN-IT1 can promote the proliferation and differentiation of SMSCs. CTTN-IT1 can be used as a new molecular marker for studying the proliferation of Hu sheep muscle cells and contribute to improving the muscle development of Hu sheep.

## Data Availability Statement

The raw data supporting the conclusions of this article will be made available by the authors, without undue reservation.

## Ethics Statement

All experimental procedures were carried out in strict accordance with the guidelines for the care and use of laboratory animals in Jiangsu Province (License Number: 45) and the recommendations of the Animal Protection and Use Committee of the Ministry of Agriculture of China (License Number: 39). The protocol was approved by the Animal Care and Use Committee at Yangzhou University.

## Author Contributions

TW and SW completed the EdU assay and wrote the draft. YL and WZ constructed the vectors and cultured cells. TW and WC analyzed the data. LW, WS, and SW designed the experiment and performed the RT-qPCR and Western blot. XL and SW gave several suggestions for manuscript modification. TW, WZ, LW, YL, XL, WC, and SW collected the sample. All authors contributed to the article and approved the submitted version.

## Conflict of Interest

The authors declare that the research was conducted in the absence of any commercial or financial relationships that could be construed as a potential conflict of interest.

## Footnotes

1 http://www.mirbase.org/

2 http://www.microrna.org/microrna/home.do

3 http://bio.sz.tsinghua.edu.cn/

4 https://cm.jefferson.edu/rna22/Interactive/
