# Supplementary figures and images for: Long Noncoding RNA (lncRNA) CTTN-IT1 Elevates Skeletal Muscle Satellite Cell Proliferation and Differentiation by Acting as ceRNA for YAP1 Through Absorbing miR-29a in Hu Sheep
Source: Front Genet. 2020 Aug 7;11:843. doi: 10.3389/fgene.2020.00843 (PMC7427492; doi:10.3389/fgene.2020.00843)

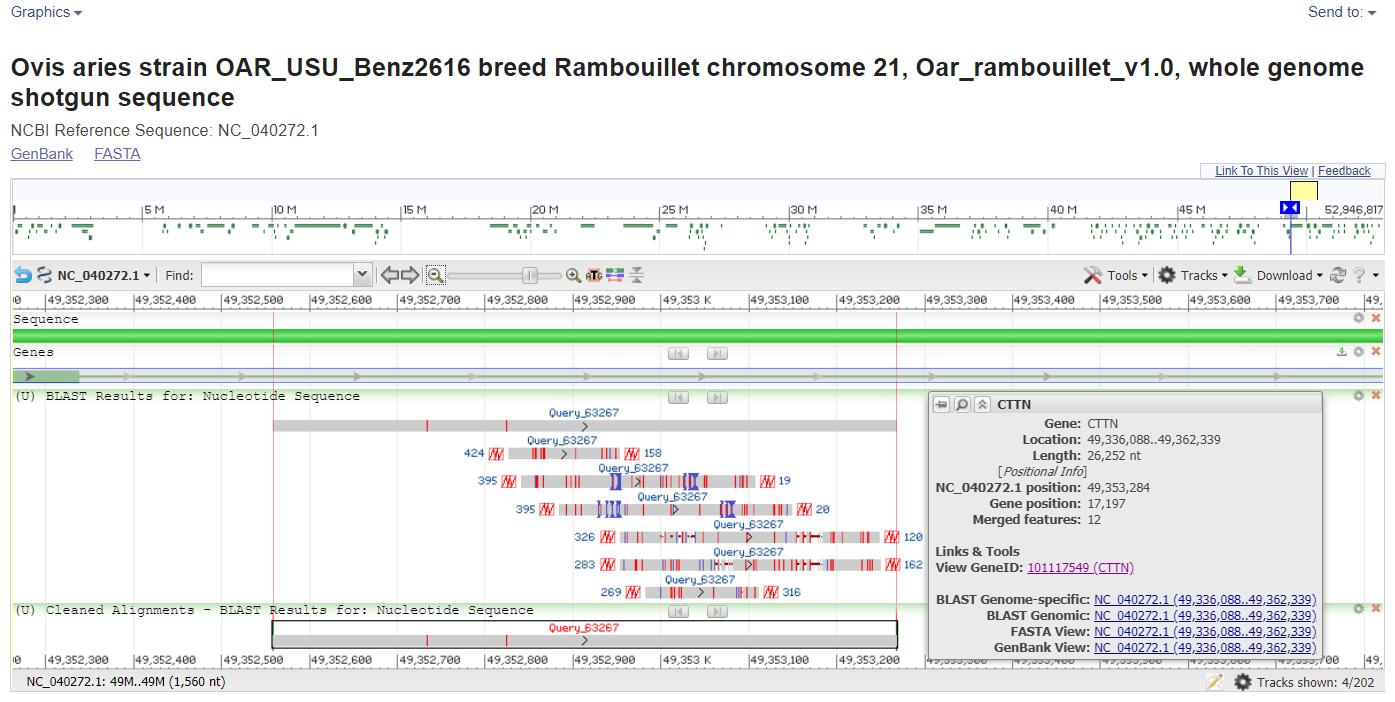

Supplement: Supplementary file 1 [file Image_1.JPEG]
