# Supplementary material for: Long Noncoding RNA (lncRNA) CTTN-IT1 Elevates Skeletal Muscle Satellite Cell Proliferation and Differentiation by Acting as ceRNA for YAP1 Through Absorbing miR-29a in Hu Sheep
Source: Front Genet. 2020 Aug 7;11:843. doi: 10.3389/fgene.2020.00843 (PMC7427492; doi:10.3389/fgene.2020.00843)
Supplement: Supplementary file 2 [file Table_1.DOCX]

**Table S1. RT-qPCR Reaction Solution System for miRNA**

| Items | Volume (μL) |
| --- | --- |
| 2×miRcute Plus miRNA Premix | 10 |
| Forward primer | 0.4 |
| Reverse primer | 0.4 |
| miRNA cDNA | 1.0 |
| double distilled H2O | 8.2 |
| Total | 20 |

**Table S2. RT-qPCR Reaction Solution System for mRNA & lncRNA**

| Items | Volume (μL) |
| --- | --- |
| 2×SYBR Premix EX Taq Ⅱ | 10.0 |
| Forward primer | 0.8 |
| Reverse primer | 0.8 |
| 50×ROX Reference Dye  cDNA | 0.4  2.0 |
| Double distilled H_2_O | 6.0 |
| Total | 20.0 |

**Table S3. Target Prediction of oar-miRNAs and *YAP1***

| miRNA | mRNA | Position | Total score | Total energy |
| --- | --- | --- | --- | --- |
| oar-miR-29a | YAP1 | 3780-3801 | 147 | -15.88 |
| oar-miR-181a | YAP1 | 2304-2326 | 159 | -16.15 |
| oar-miR-541-3p | YAP1 | 442-463 | 140 | -20.31 |
| oar-miR-200 | YAP1 | 1020-1041 | 151 | -15.00 |
