# Supplementary material for: Long Noncoding RNA (lncRNA) CTTN-IT1 Elevates Skeletal Muscle Satellite Cell Proliferation and Differentiation by Acting as ceRNA for YAP1 Through Absorbing miR-29a in Hu Sheep
Source: Front Genet. 2020 Aug 7;11:843. doi: 10.3389/fgene.2020.00843 (PMC7427492; doi:10.3389/fgene.2020.00843)
Supplement: Supplementary file 4 [file Data_Sheet_2.DOCX]

**>YAP1 Wild Binding Site 535 bp [organism=Ovis aries]**

GCCTACAATTTGCCATTAAGCCACAAATTAAGATCTCATCTTATATATCAGCAGAGTAGCTTTAGATTsAGGGGAAAGGGTGGGAAAATGGGAGGGGGATTGTGAAGATTTAGTGGGACCTTGATAGAGAACTTTATAAGCTTCTTTTTCTTCAATAAAAACTTGTCTTGTATATTGCTGTCATTAAAAGCAGTTGTTCCTAAAATTTCAATCACTTAAGTACACCCACAAAACAAAAATACGGAGTTCTTCATTCCCCCTCGATTTGGATTTACCCAGTTATACCTCAGTGTTGTGGCAGCACCGTGATGTCTGAAGGACATGGTGCTTTGACCTAATCGTAACCGTTGTACTGACCTGAAGGAGACCTAAGCCTTTCTCTTTCTGAATTTGAATCACAGTCTTGATGTGGTCTTTCTTGTTTTTTGTCCTTGTTCCTAATGTAAAAGTGTTTAACTGCTTCTTGGTTGTATTGGGTGGCATT

**>YAP1 Mutant Site [organism=Ovis aries]**

GCCTACAATTTGCCATTAAGCCACAAATTAAGATCTCATCTTATATATCAGCAGAGTAGCTTTAGATTAGGGGAAAGGGTGGGAAAATGGGAGGGGGATTGTGAAGATTTAGTGGGACCTTGATAGAGAACTTTATAAGCTTCTTTTTCTTCAATAAAAACTTGTCTTGTATATTGCTGTCATTAAAAGCAGTTGTTCCTAAAATTTCAATCACTTAAGTACACCCACAAAACAAAAATACGGAGTTCTTCATTCCCCCTCGATTTGGATTTACCCAGTTATACCTCAGTGTTGTGGCAGCACCGTGtgGatTGAAGGACATGGTGCTTTGACCTAATCGTAACCGTTGTACTGACCTGAAGGAGACCTAAGCCTTTCTCTTTCTGAATTTGAATCACAGTCTTGATGTGGTCTTTCTTGTTTTTTGTCCTTGTTCCTAATGTAAAAGTGTTTAACTGCTTCTTGGTTGTATTGGGTGGCATT

NOTE: highlighted lowercase letters indicate mutation sites.

**>CTTN-IT1 Binding Site 710 bp [organism=Ovis aries]**

GTCTCTGGAGCGGGCGCCTGGCCTCCCTGGGCCTCCCCCGGCAGCTCTCGCTGTTGACATGGCTGCTGTGACTGGAATAAGCCGCCTGCCTGCCTCTGAGGCCGGGAGAAAGGTGTTCGGTTGAGTGTGATTACTGGCCTTGGACCAGAGGGGCACAGGGCAGGCGTGTCCACATGTCTTTTGGGTCTCAGTTGTTGGAGTGTTCTTTGGGGTTTTGTTTTTGATGACCCTGTCTGCGTGTAACTCCACAACAAAGCAGACAGTACCTACAGGAAGCTGGAATCACAGAGGGTCTGGGTATATGCTCACATTCAGGAAGCTGGAATCACAGGGGTTCTGGGTGGTACTCAGGTTCAGGAAGCTGGAATCACGGGTTCTGGGTG

TGTGCTCAGGTACACAGGAGGGTCTGGGTGGTGC^1^TCAGATTCAGGAAGCTGGAATCACAGGAGGGTCTGGTTGTGTGCTGAGGTTCAGGAAGCTGGAATCACAGGGGTTCTGGGTGGTGCTCAGGTTCAGGAAGCTGGAATCACAGGAGGGTCTGAGTGGTGC^2^TGAGGTTCAGGAAGCTGGAATCACTGGGGTTCTGGGTGTGTGCTCAGGTACGCAGGAGGGTCTGGGTGGTGCTCAGGTACACAGGAGGGACCGGGTGGTGC^3^TCAGGTTCAGGAAGCTGGAATCCCAGGGGCTTTGGGTGGTGCTGGGAGTTGAGATGAAGAGTG

**>CTTN-IT1 Mutant Site [organism=Ovis aries]**

GTCTCTGGAGCGGGCGCCTGGCCTCCCTGGGCCTCCCCCGGCAGCTCTCGCTGTTGACATGGCTGCTGTGACTGGAATAAGCCGCCTGCCTGCCTCTGAGGCCGGGAGAAAGGTGTTCGGTTGAGTGTGATTACTGGCCTTGGACCAGAGGGGCACAGGGCAGGCGTGTCCACATGTCTTTTGGGTCTCAGTTGTTGGAGTGTTCTTTGGGGTTTTGTTTTTGATGACCCTGTCTGCGTGTAACTCCACAACAAAGCAGACAGTACCTACAGGAAGCTGGAATCACAGAGGGTCTGGGTATATGCTCACATTCAGGAAGCTGGAATCACAGGGGTTCTGGGTGGTACTCAGGTTCAGGAAGCTGGAATCACGGGTTCTGGGTG

TGTGCTCAGGTACACAGGAGGGTCTGGGattctC^1^TCAGATTCAGGAAGCTGGAATCACAGGAGGGTCTGGTTGTGTGCTGAGGTTCAGGAAGCTGGAATCACAGGGGTTCTGGGTGGTGCTCAGGTTCAGGAAGCTGGAATCACAGGAGGGTCTGAGTaactt^2^TGAGGTTCAGGAAGCTGGAATCACTGGGGTTCTGGGTGTGTGCTCAGGTACGCAGGAGGGTCTGGGTGGTGCTCAGGTACACAGGAGGGACCGGGaatctC^3^TCAGGTTCAGGAAGCTGGAATCCCAGGGGCTTTGGGTGGTGCTGGGAGTTGAGATGAAGAGTG

NOTE: 1,2 and 3 indicate the mutant site of Mutant-1, Mutant-2, and Mutant-3.
